# Effects of transcranial direct current stimulation on brain activity and cortical functional connectivity in children with autism spectrum disorders

**DOI:** 10.3389/fpsyt.2024.1407267

**Published:** 2024-05-15

**Authors:** Jiannan Kang, Yuqi Li, Shuaikang Lv, Pengfei Hao, Xiaoli Li

**Affiliations:** ^1^ College of Electronic & Information Engineering, Hebei University, Baoding, China; ^2^ State Key Laboratory of Cognitive Neuroscience and Learning, Beijing Normal University, Beijing, China

**Keywords:** autism spectrum disorder, electroencephalogram, transcranial direct current stimulation, sLORETA, lagged phase synchronization

## Abstract

**Introduction:**

Transcranial direct current stimulation (tDCS) has emerged as a therapeutic option to mitigate symptoms in individuals with autism spectrum disorder (ASD). Our study investigated the effects of a two-week regimen of tDCS targeting the left dorsolateral prefrontal cortex (DLPFC) in children with ASD, examining changes in rhythmic brain activity and alterations in functional connectivity within key neural networks: the default mode network (DMN), sensorimotor network (SMN), and dorsal attention network (DAN).

**Methods:**

We enrolled twenty-six children with ASD and assigned them randomly to either an active stimulation group (n=13) or a sham stimulation group (n=13). The active group received tDCS at an intensity of 1mA to the left DLPFC for a combined duration of 10 days. Differences in electrical brain activity were pinpointed using standardized low-resolution brain electromagnetic tomography (sLORETA), while functional connectivity was assessed via lagged phase synchronization.

**Results:**

Compared to the typically developing children, children with ASD exhibited lower current source density across all frequency bands. Post-treatment, the active stimulation group demonstrated a significant increase in both current source density and resting state network connectivity. Such changes were not observed in the sham stimulation group.

**Conclusion:**

tDCS targeting the DLPFC may bolster brain functional connectivity in patients with ASD, offering a substantive groundwork for potential clinical applications.

## 1 Introduction

Autism Spectrum Disorder (ASD) encompasses a diverse range of neurodevelopmental conditions characterized by challenges in social communication, along with the presence of stereotyped, repetitive behaviors, and restricted interests. These symptoms, stemming from atypical nervous system development, emerged in early childhood, yet the pathogenesis remained elusive (1). Currently, interventions for children with ASD primarily consist of conventional rehabilitation therapies aimed at altering psychological states and rectifying abnormal behaviors through psychological and behavioral means to facilitate treatment outcomes. Nonetheless, targeted therapies for ASD are still under active investigation.

The emergence of non-invasive stimulation techniques in autism research has propelled transcranial direct current stimulation (tDCS) to the forefront. tDCS delivers a steady stream of electrical current to the cerebral cortex, modulating cortical excitability—it is both safe and non-invasive (2). tDCS influences neuronal excitability and plasticity by administering electrical currents and offers benefits such as mild stimulation intensity, affordability, and a reduced risk of adverse effects. Its application spans cognitive impairment, depression, and Alzheimer’s disease treatments and rehabilitation research (3, 4) and showed promise for addressing cognitive deficits associated with neurological conditions (5). Importantly, tDCS does not require prolonged stillness from children, making it particularly suitable for children with ASD who may exhibit low functioning and hyperactivity. The modality also includes sham stimulation settings, accommodating the demands of clinical research. Cumulative evidence suggests that applying tDCS to the frontal cortex can markedly influence brain activities, including attention (6), learning (6–8), memory (7–9), and vigilance (10). These profound effects position tDCS as a potential therapeutic modality for ASD, although the efficacy of tDCS in children with ASD requires further exploration.

Resting-state Electroencephalogram (EEG) can reveal intrinsically connected functional networks, unobscured by the demands of cognitive tasks. While connectivity studies in EEG have traditionally relied on measurements between electrode pairs, this approach potentially suffers from volume conduction issues (11) and imprecise anatomical location estimates, assuming a two-dimensional construct rather than a true three-dimensional framework. Conversely, three-dimensional analysis utilizing EEG may furnish more intricate and accurate insights.

EEG source localization aims to identify sources of neural activity in the brain through potential differences between EEG electrodes on the scalp. An important development was the introduction of anatomical constraints on the head to facilitate the problem of EEG source location (12, 13). Further developments introduced physiological constraints of cortical sources to facilitate the solution of distributed source imaging (14). This prior constraint greatly improves the solvability and precision of brain source location. The distributed source model is based on thousands of orientationally fixed dipole sources that are regularly distributed in a three-dimensional (3D) brain space or cortical surface. The activation of the source is described as a current density distribution. One advantage of the distributed model is that there is no need to make a prior assumption about the number of active dipoles under a particular scalp voltage diagram. The minimum norm algorithm was proposed in 1994 to estimate the source current distribution as an alternative to single or multiple dipole models. Since the minimum norm tends to mislocate deeper sources, the weighted minimum norm method can compensate for this with a weighted matrix. Low resolution electromagnetic Tomography (LORETA) aims to find the smoothest minimum norm solution to the EEG activity distribution, standardized LORETA (sLORETA) standardizes the current density estimate given by the minimum norm by using the covariance of the resolution matrix and is able to achieve zero error localization. We can examine EEG data through the lens of current source density measurements, made possible by standardized low-resolution electromagnetic tomography (sLORETA). This methodology synergized the high temporal resolution of EEG with the spatial localization of cerebral electrical activities (15). Correlation analysis revealed augmented short-range connectivity between regions implicated in the mirror neuron system and social perception networks (16). A previous magnetoencephalography investigation into ASD employed source localization to assess resting-state networks (RSN), including the default mode and salience networks (17). The findings associated reductions in gamma-band connectivity within the DMN and between the DMN and salience networks with ASD severity, particularly concerning social communication and interaction capacities. Historical focus has been scant on the sensorimotor network (SMN), which underlies core ASD symptoms due to atypical sensorimotor and perceptual processes (18–24). Moreover, studies regarding the functional brain networks sustaining attentional faculties are rare. The dorsal attention network (DAN) orchestrates goal-driven top-down control processes (25) and deficits in the connectivity within this network may underpin the attentional challenges characteristic of ASD (26, 27).

Hence, based on the above evidence, we conducted a shame-controlled trial to investigate the efficacy of tDCS in children with ASD. This study hypothesized that (1) significant differences of current source density in delta, theta, alpha, and beta rhythm in the DMN, SMN, and DAN would be observed between children with ASD and typically developing (TD) children. (2) active tDCS stimulation would increase current source density in the above described resting state networks and rhythms in the brain of children with ASD. (3) active tDCS stimulation would enhance functional connectivity (lagged phase synchronization) of resting-state networks in the brain of children with ASD.

## 2 Materials and methods

### 2.1 Subject information

Our study cohort consisted of 13 subjects (11 males and 2 females; mean age ± SD: 5.6 ± 1.8 years) undergoing 10 sessions of tDCS treatment and a matched control group of 13 subjects (11 males and 2 females; mean age ± SD: 5.4 ± 2.2 years). All subjects were diagnosed with ASD by qualified psychiatrists according to the PEP-III (28) and DSM-IV-TR criteria (29). Informed consent was obtained in written form, with full comprehension of the experimental protocols provided to the parents beforehand. The clinical trial adhered to the Declaration of Helsinki and received approval from the Ethics Committee of the Affiliated Hospital of Hebei University.

Inclusion criteria for ASD subjects included (1) confirmation of autism by a certified child psychiatrist, (2) an age range of 4–6 years, and (3) provision of written informed consent. Exclusion criteria encompassed (1) a history of neurological diseases such as epilepsy, brain injury, or neurosurgery; (2) concurrent or prior use of antipsychotic or antiepileptic medications; and (3) previous exposure to tDCS, repetitive transcranial magnetic stimulation (rTMS), or neurofeedback interventions prior to this study.

### 2.2 EEG data acquisition

EEG recordings were conducted in an electromagnetically isolated environment, with subjects seated comfortably and wearing EEG caps, eyes open throughout the procedure. A parent and a trained expert were present to supervise the children and ensure the integrity of the data. Participants were gently encouraged to minimize blinking, with unmanageable eye movement artifacts subsequently removed during data preprocessing. Recordings lasted approximately 5-10 minutes and utilized a 128-channel HydroCel geodesic sensor net (Electrical Geodesics, Inc., Eugene, Oregon, USA), maintaining electrode impedance below 50 kΩ. Data was sampled at 1000 Hz using Cz as the reference electrode.

### 2.3 Data preprocessing

Data processing was performed off-line with Matlab R2016a and EEGlab V13.5.4b. Post-downsampling to 200 Hz, a 1–45 Hz band-pass filter was employed. Independent component analysis (ICA) (30) was used to remove artifacts linked to eye blinks, muscular movements, and electromyography. Data were then re-referenced to an average reference, and 19 electrodes were selected for further analysis according to the 10-20 system. EEG recordings for each participant were segmented into 24-second epochs, which were further divided into 3-second segments (8 per subject), and were systematically arranged within respective participant folders.

### 2.4 Stimulation procedure

Stimulation was performed using a 1 mA direct current delivered via a battery-powered stimulator (JX-tDCS-1, Huahengjingxing New Medical Technology Co., Ltd., Nanchang, China), with a consistent impedance below 10 kΩ between saline-soaked sponge electrodes (7 × 4.5 cm). For both active and sham stimulation protocols, the anode was positioned on the left DLPFC, while the cathode was placed on the right supraorbital area. In the active tDCS condition, the current was gradually raised to 1 mA over 30 seconds, maintained for 20 minutes, and then decreased to zero within 30 seconds. The sham tDCS mirrored the electrode placement of the active stimulation but involved current flow merely for the initial 20 seconds.

### 2.5 Source location analysis and statistical nonparametric mapping

We utilized the sLORETA/eLORETA software package to estimate current source densities across 6239 cortical and hippocampal gray matter voxels, with a spatial resolution of 5 mm (15, 31). The voxel-by-voxel sLORETA data in various frequency bands—delta, theta, alpha, and beta—underwent analysis to discern differences between the ASD and TD groups, as well as within the active and sham tDCS-treated cohorts. The Statistical Nonparametric Mapping (SnPM) technique employed 5000 randomized, voxel-specific paired t-tests to perform comparative assessments. Prior to statistical evaluation, sLORETA images were logarithmically transformed to address non-specific regional confounds (32). Corrections for multiple comparisons were applied to the pooled SnPM results (33, 34). T-thresholds corresponding to a significance level of p< 0.05 were calculated using statistical utilities from sLORETA.

### 2.6 Functional connectivity analysis

#### 2.6.1 Region of interest

We derived ROIs for the SMN, DMN, and DAN following the framework established by Wantzen et al. (35), totaling 21 key areas. Each ROI encapsulated all gray matter voxels within a 10 mm radius, averaging the logarithmically transformed current densities across the voxels assigned to each ROI. Detailed names and corresponding coordinates of all regions are presented in **Table 1**.

**Table 1:** Regions of interest.

	Regions	MNI coordinates
DMN	Right Superior temporal gyrus	41 -60 29
	Left Superior temporal gyrus	-41 -60 29
	Medial frontal gyrus	0 49 18
	Posterior cingulate cortex	0 -52 26
SMN	Right Precentral gyrus	34 -32 59
	Left Postcentral gyrus	-44 -30 58
	Medial frontal gyrus	-4 -33 66
	Left Transverse temporal gyrus	-56 -23 12
	Right Transverse temporal gyrus	55 -23 12
DAN	Right Middle frontal gyrus	22 -8 54
	Left Middle frontal gyrus	-22 -8 54
	Right lnferior parietal lobule	34 -38 44
	Left lnferior parietal lobule	-34 -38 44
	Right Precuneus	18 -69 51
	Left Precuneus	-18 -69 51
	Right Middle temporal gyrus	51 -64 -2
	Left Middle temporal gyrus	-51 -64 -2
	Right Precuneus-superior parietal lobule	8 -63 57
	Left Superior parietal lobule	-8 -63 57
	Right lnferior frontal gyrus	49 3 34
	Left Inferior frontal gyrus	-49 3 34

#### 2.6.2 Functional connectivity

Our study employed sLORETA to compute lagged phase synchronization for the functional connectivity analysis. This approach assesses signal similarity in the frequency domain, accounting for volume conduction and other non-physiological influences, thus providing a corrected phase synchronization value based on a normalized Fourier transform (22, 36). We assessed connectivity within networks by comparing lagged phase synchronization among ROIs for artifact-free EEG segments across the delta, theta, alpha, and beta bands. The log-transformed data underwent analysis using one-tailed t-statistics, with corrections for multiple comparisons achieved through a non-parametric permutation method involving 5000 randomizations. The derived t-thresholds reflect the designated significance level (p< 0.05) for functional connectivity within the SMN, DAN, and DMN networks.

## 3 Results

### 3.1 Source location results

#### 3.1.1 Comparison of current source density between ASD group and TD group

**Figure 1:**
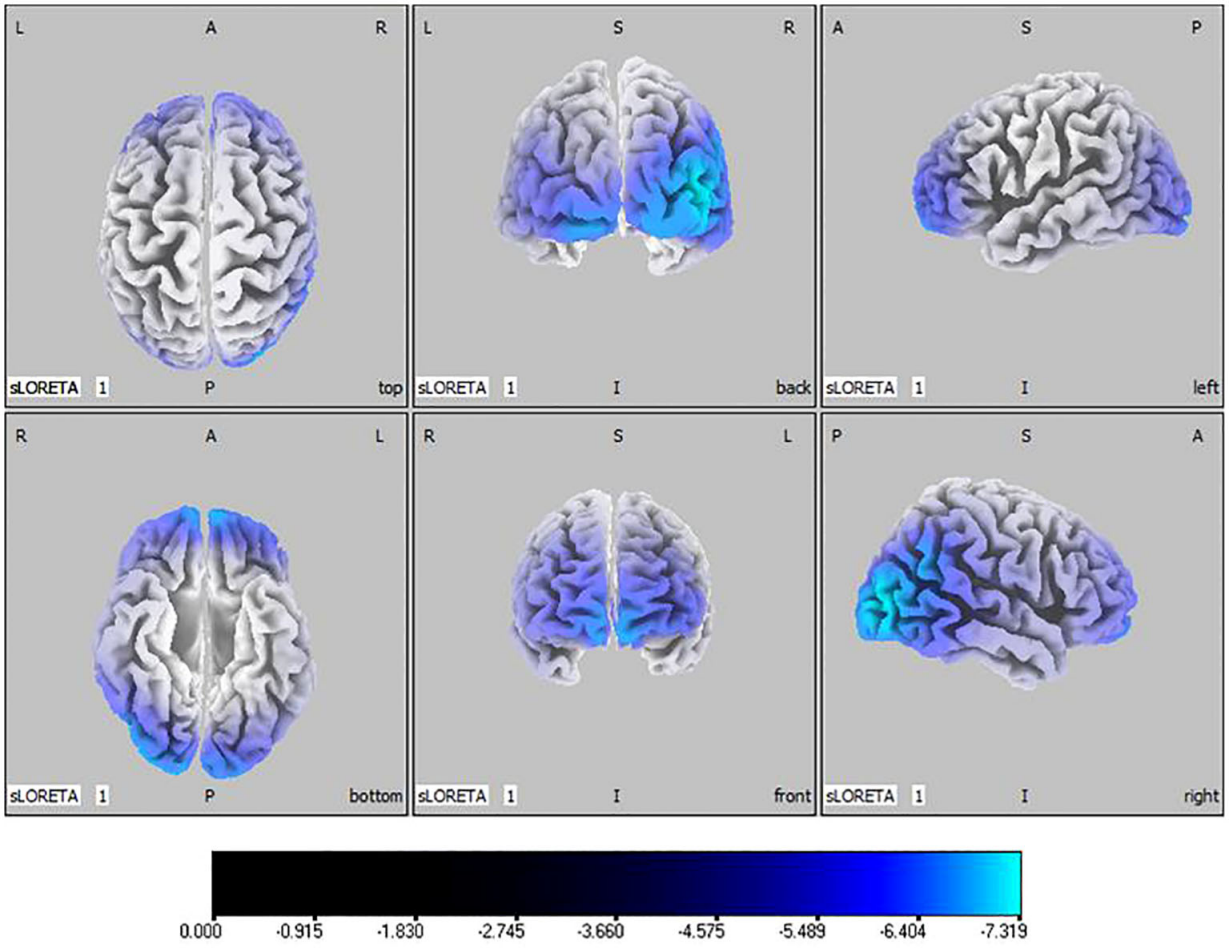
Source localization differences between ASD and TD groups in delta band: the blue areas denote significant reductions in current source density within the ASD group (p< 0.05). Orientational abbreviations indicate the following: L for left hemisphere, R for right hemisphere, A for anterior (frontal) regions, P for posterior (rear) regions, and S for superior (upper) regions of the brain.

**Figure 2:**
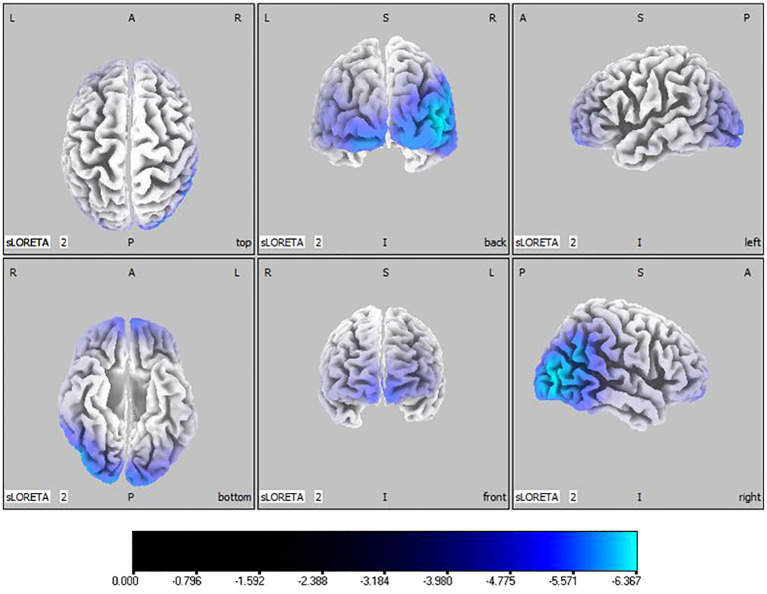
Source localization differences between ASD and TD groups in theta band: the blue areas denote significant reductions in current source density within the ASD group (p< 0.05). Orientational abbreviations indicate the following: L for left hemisphere, R for right hemisphere, A for anterior (frontal) regions, P for posterior (rear) regions, and S for superior (upper) regions of the brain.

**Figure 3:**
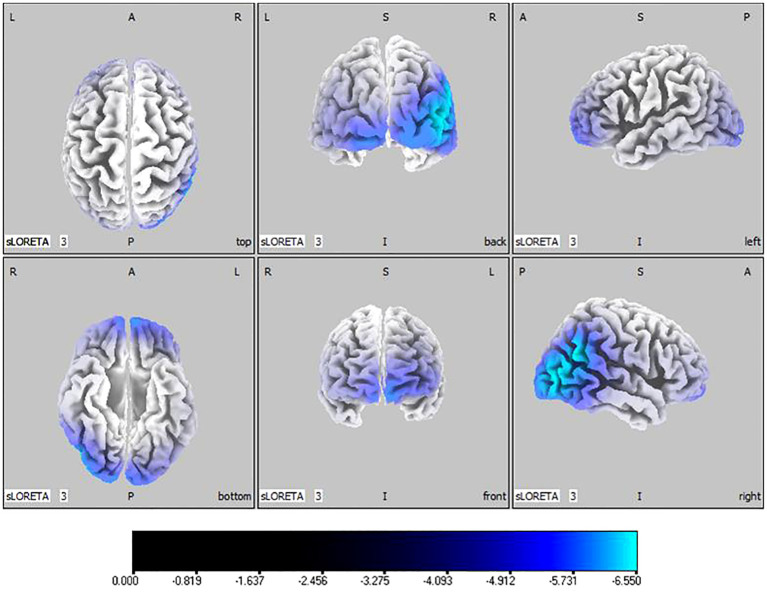
Source localization differences between ASD and TD groups in alpha band: the blue areas denote significant reductions in current source density within the ASD group (p< 0.05). Orientational abbreviations indicate the following: L for left hemisphere, R for right hemisphere, A for anterior (frontal) regions, P for posterior (rear) regions, and S for superior (upper) regions of the brain.

**Figure 4:**
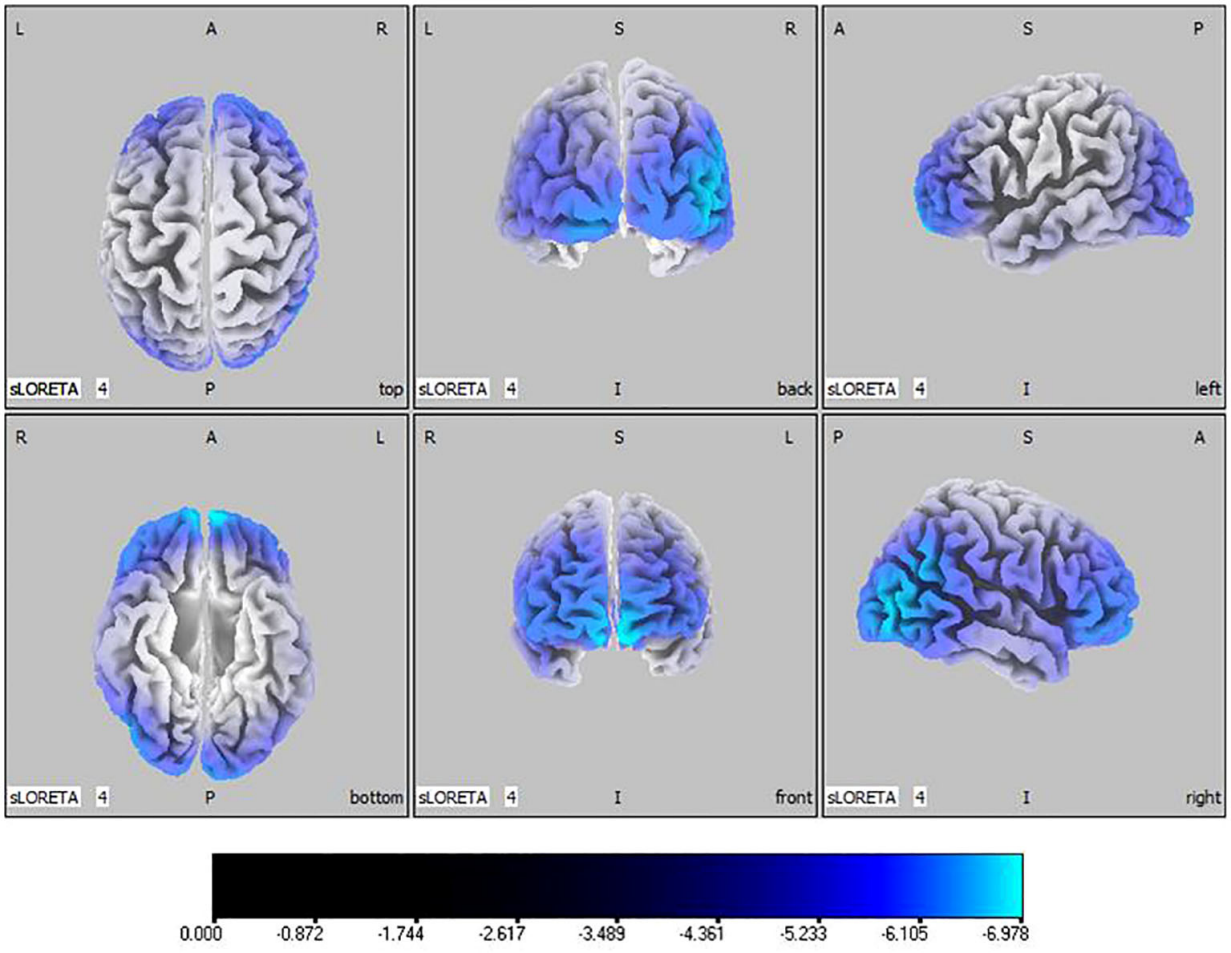
Source localization differences between ASD and TD groups in beta band: the blue areas denote significant reductions in current source density within the ASD group (p< 0.05). Orientational abbreviations indicate the following: L for left hemisphere, R for right hemisphere, A for anterior (frontal) regions, P for posterior (rear) regions, and S for superior (upper) regions of the brain.

Compared with the TD group, the source localization results across the delta, theta, alpha, and beta bands in the ASD group indicated that the current source density was significantly reduced. Among them, the number of voxels with significant differences in delta band is 5418, the number of voxels with significant differences in theta band is 3603, the number of voxels with significant differences in alpha band is 3854, the number of voxels with significant differences in beta band is 5425. Specifically, delta band analysis revealed a notable decrease in the prefrontal and occipital regions, peaking in the middle occipital gyrus with a maximum value of ( $t_{max}=$ - 7 .319, xyz= 45, -85, 10) as depicted in **Figure 1**. Theta band observations showed substantial reduction in the occipital lobe, with the most significant value ( ${\text{t}}_{\text{max}}=$ -6.367, xyz= 50, -80, 10) situated in the middle temporal gyrus, illustrated in **Figure 2**. In the alpha band, reductions in current source density were eminent in the left frontal lobe, occipital lobe, and right temporal lobe, with the middle temporal gyrus showing the greatest difference ( $t_{max}=$ -6.550, xyz= 50, -80, 10), shown in **Figure 3**. Beta band results exhibited considerable decreases across the frontal, occipital, right temporal, and parietal lobes, with the middle frontal gyrus reflecting the highest discrepancy ( $t_{max}=$ -6.978, xyz= -5, 65, -15), demonstrated in **Figure 4**.

#### 3.1.2 Comparison of current source density before and after tDCS stimulation

**Figure 5:**
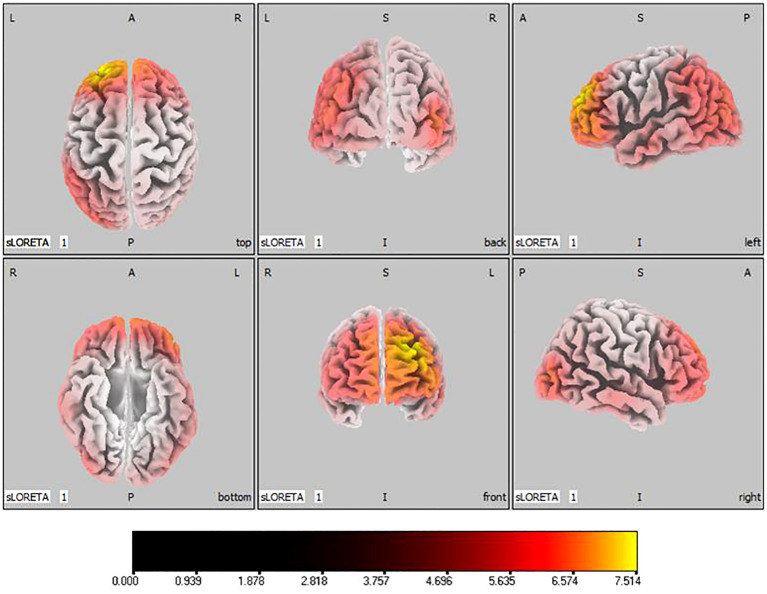
Source localization differences between pre-stimulation and post-stimulation groups in delta band. Red areas indicate the range in the post-stimulation group where the current source density increased significantly (p< 0.05). Orientational abbreviations indicate the following: L for left hemisphere, R for right hemisphere, A for anterior (frontal) regions, P for posterior (rear) regions, and S for superior (upper) regions of the brain.

**Figure 6:**
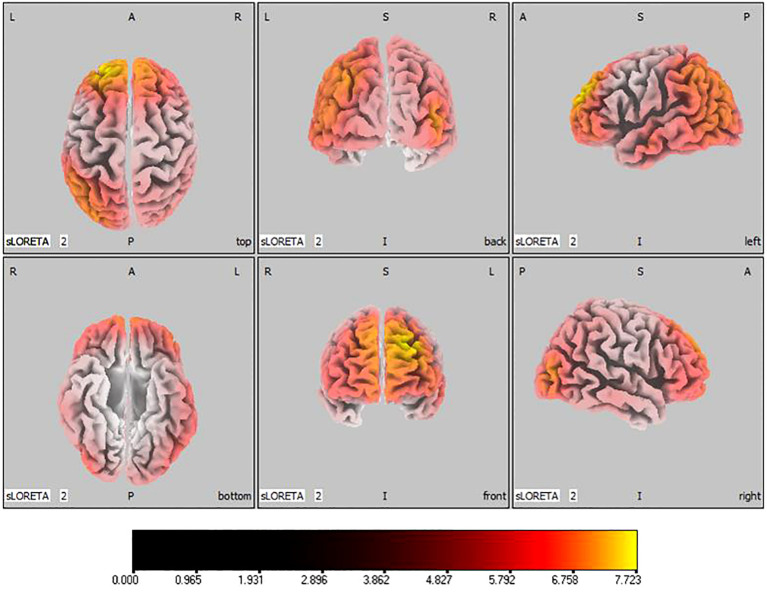
Source localization differences between pre-stimulation and post-stimulation groups in theta band. Red areas indicate the range in the post-stimulation group where the current source density increased significantly (p< 0.05). Orientational abbreviations indicate the following: L for left hemisphere, R for right hemisphere, A for anterior (frontal) regions, P for posterior (rear) regions, and S for superior (upper) regions of the brain.

**Figure 7:**
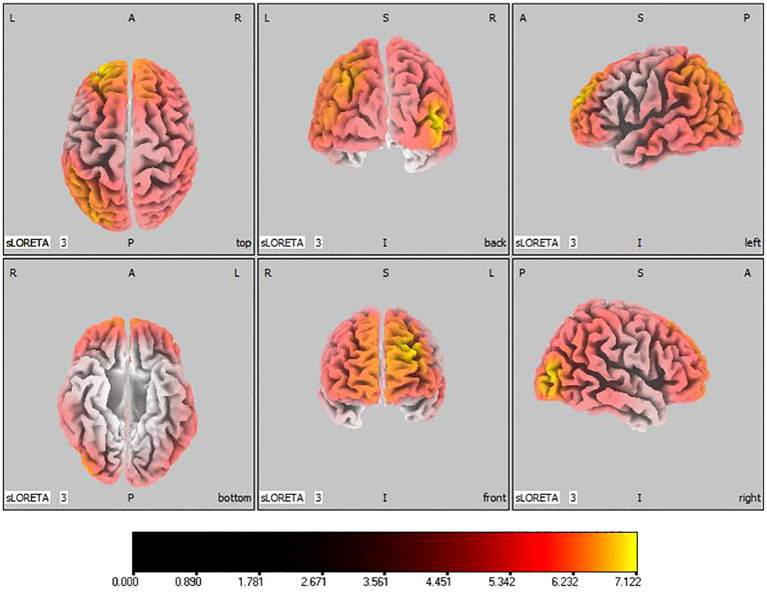
Source localization differences between pre-stimulation and post-stimulation groups in alpha band. Red areas indicate the range in the post-stimulation group where the current source density increased significantly (p< 0.05). Orientational abbreviations indicate the following: L for left hemisphere, R for right hemisphere, A for anterior (frontal) regions, P for posterior (rear) regions, and S for superior (upper) regions of the brain.

**Figure 8:**
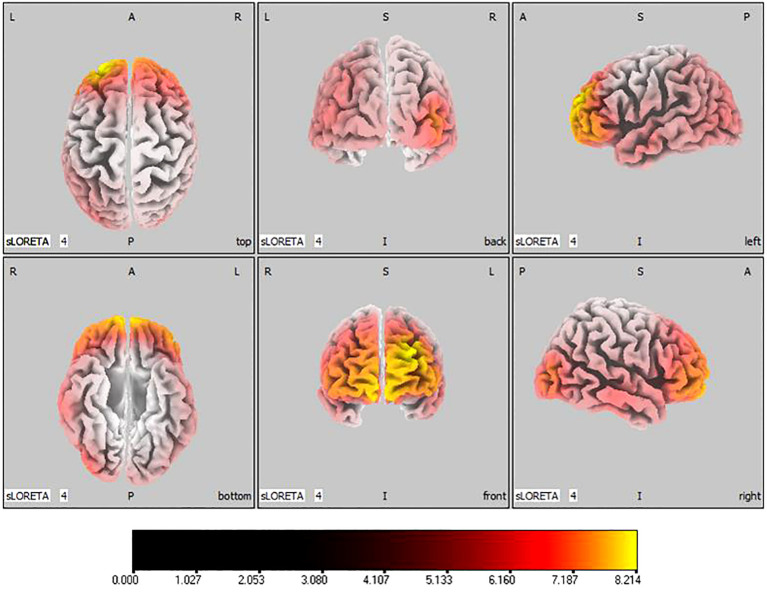
Source localization differences between pre-stimulation and post-stimulation groups in beta band. Red areas indicate the range in the post-stimulation group where the current source density increased significantly (p< 0.05). Orientational abbreviations indicate the following: L for left hemisphere, R for right hemisphere, A for anterior (frontal) regions, P for posterior (rear) regions, and S for superior (upper) regions of the brain.

In the active stimulation group, post-treatment source localization in the delta, theta, alpha, and beta bands indicated meaningful increases in current source density. Among them, the number of voxels with significant differences in delta band is 5910, the number of voxels with significant differences in theta band is 6039, the number of voxels with significant differences in alpha band is 6129, the number of voxels with significant differences in beta band is 5940. The delta band exhibited pronounced enhancements primarily in the frontal lobe, left parietal temporal lobe, and right occipital lobe, with the superior frontal gyrus presenting the highest increase ( $t_{max}=$ 7.514, xyz= -20, 55, 35), as shown in **Figure 5**. Theta band measurements displayed significant advancements in the frontal, parietal, occipital, and left temporal lobes, centered again in the superior frontal gyrus ( $t_{max}=$ 7.723, xyz= -20, 55, 35), represented in **Figure 6**. Sizable improvements in the alpha band spanned the frontal lobe, central area, parietal lobe, occipital lobe, and temporal lobe domains, with the middle occipital gyrus manifesting the foremost elevation ( $t_{max}=$ 7.122, xyz= 45, -85, 5), shown in **Figure 7**. For the beta band, notable enhancements were located in the frontal, temporal and right occipital regions, with the peak increase recorded in the superior frontal gyrus ( $t_{max}=$ 8.214, xyz= -25, 55, 30), exhibited in **Figure 8**. The sham stimulation group did not demonstrate significant changes.

### 3.2 Functional connectivity within brain networks

**Figure 9:**
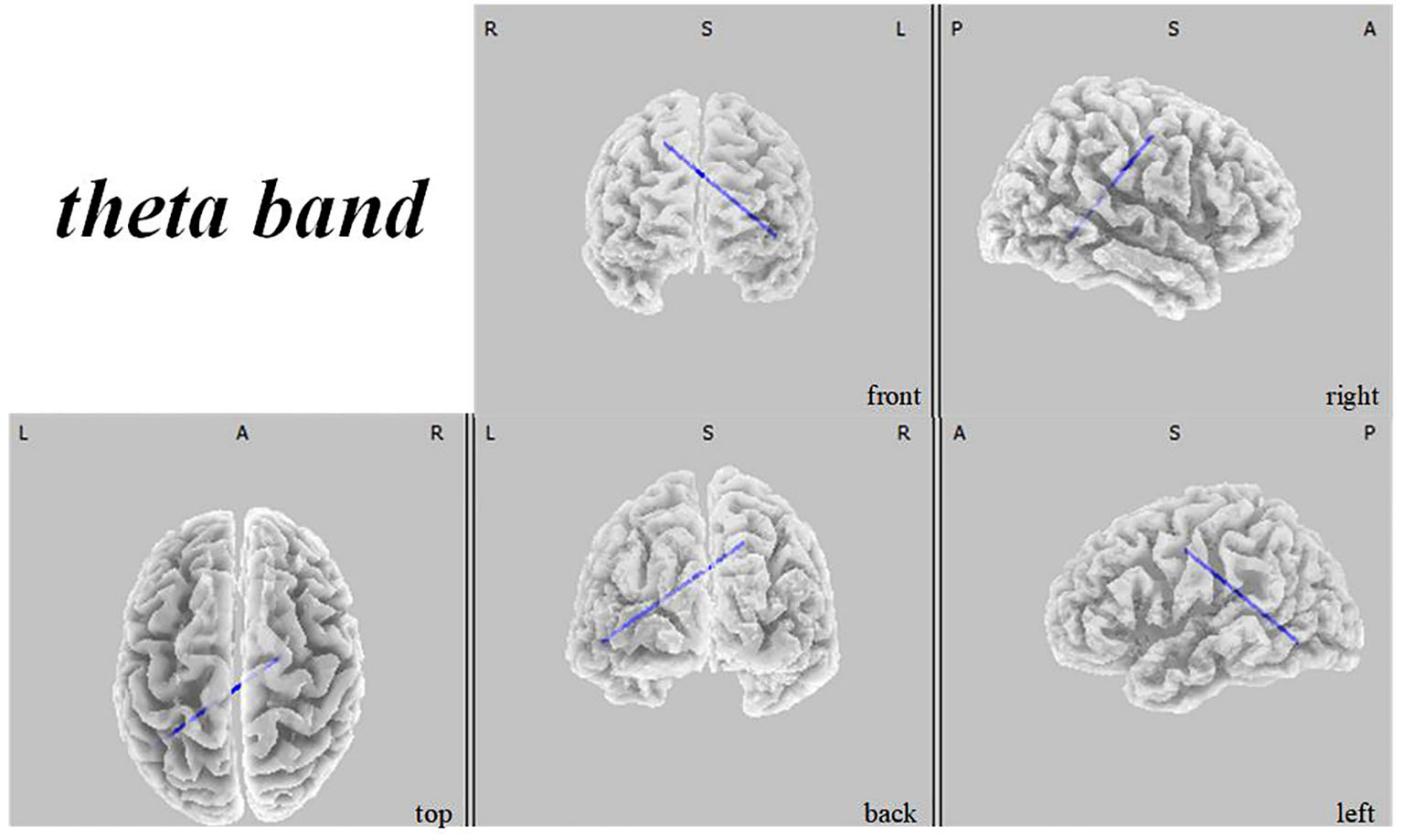
Connectivity variation diagram highlighting significant differences between ASD and TD groups. Blue lines represent a significant reduction in lagged phase synchronization within DAN for the pre-stimulation group as compared to the post-stimulation group. Orientational abbreviations indicate the following: L for left hemisphere, R for right hemisphere, A for anterior (frontal) regions, P for posterior (rear) regions, and S for superior (upper) regions of the brain.

The results showed that functional connectivity in the brains of children in the ASD group was significantly reduced in DAN compared to the TD group. It is embodied between Left Middle temporal gyrus and Right lnferior frontal gyrus ( t =-3.530, p< 0.024) in theta band, as seen in **Figure 9**. There was no significant difference in functional connectivity between SMN and DMN.

**Figure 10:**
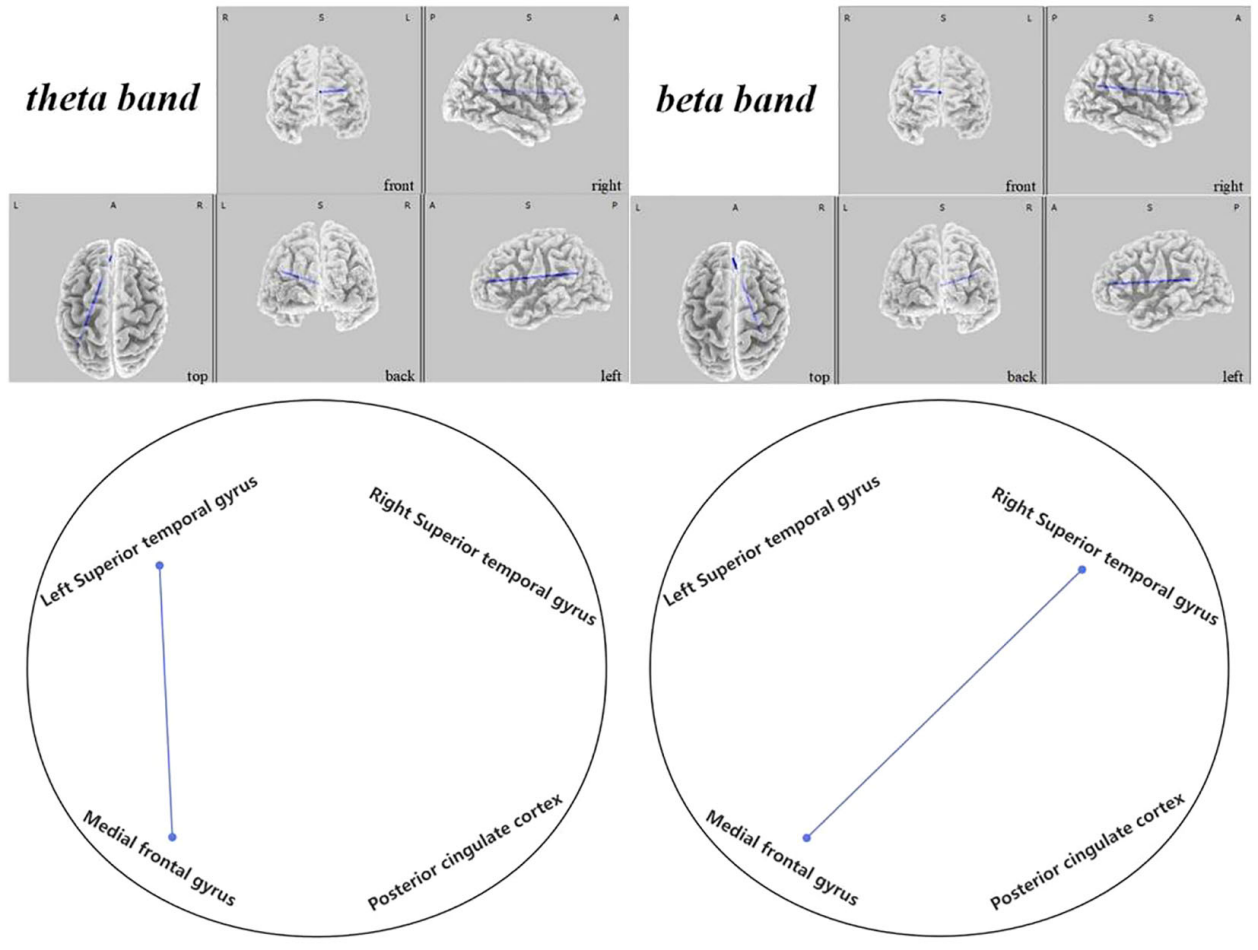
Connectivity variation diagram highlighting significant differences between pre-stimulation and post-stimulation groups. Blue lines represent a significant reduction in lagged phase synchronization within DMN for the pre-stimulation group as compared to the post-stimulation group. Orientational abbreviations indicate the following: L for left hemisphere, R for right hemisphere, A for anterior (frontal) regions, P for posterior (rear) regions, and S for superior (upper) regions of the brain.

**Figure 11:**
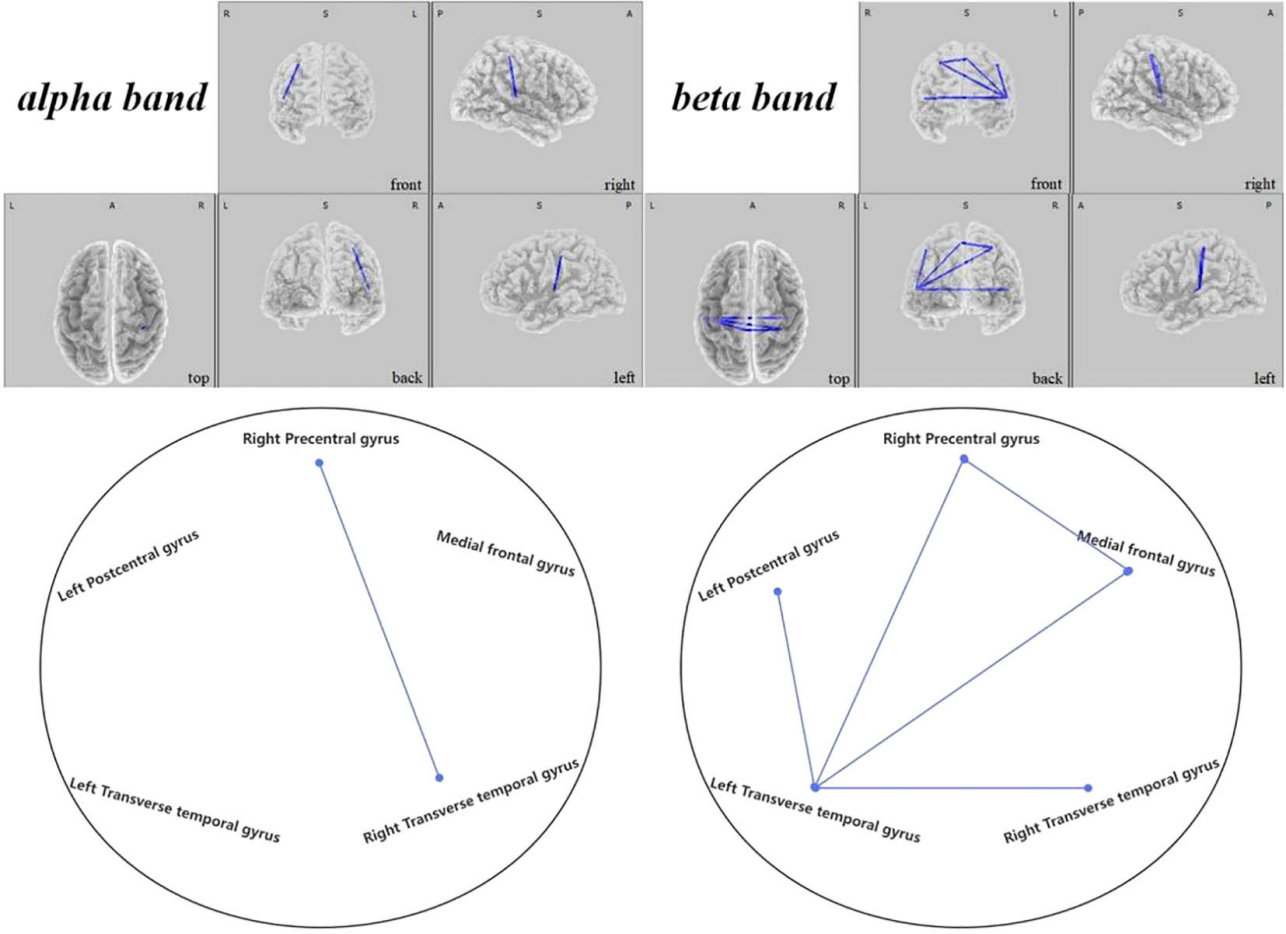
Connectivity variation diagram highlighting significant differences between pre-stimulation and post-stimulation groups. Blue lines represent a significant reduction in lagged phase synchronization within SMN for the pre-stimulation group as compared to the post-stimulation group. Orientational abbreviations indicate the following: L for left hemisphere, R for right hemisphere, A for anterior (frontal) regions, P for posterior (rear) regions, and S for superior (upper) regions of the brain.

**Figure 12:**
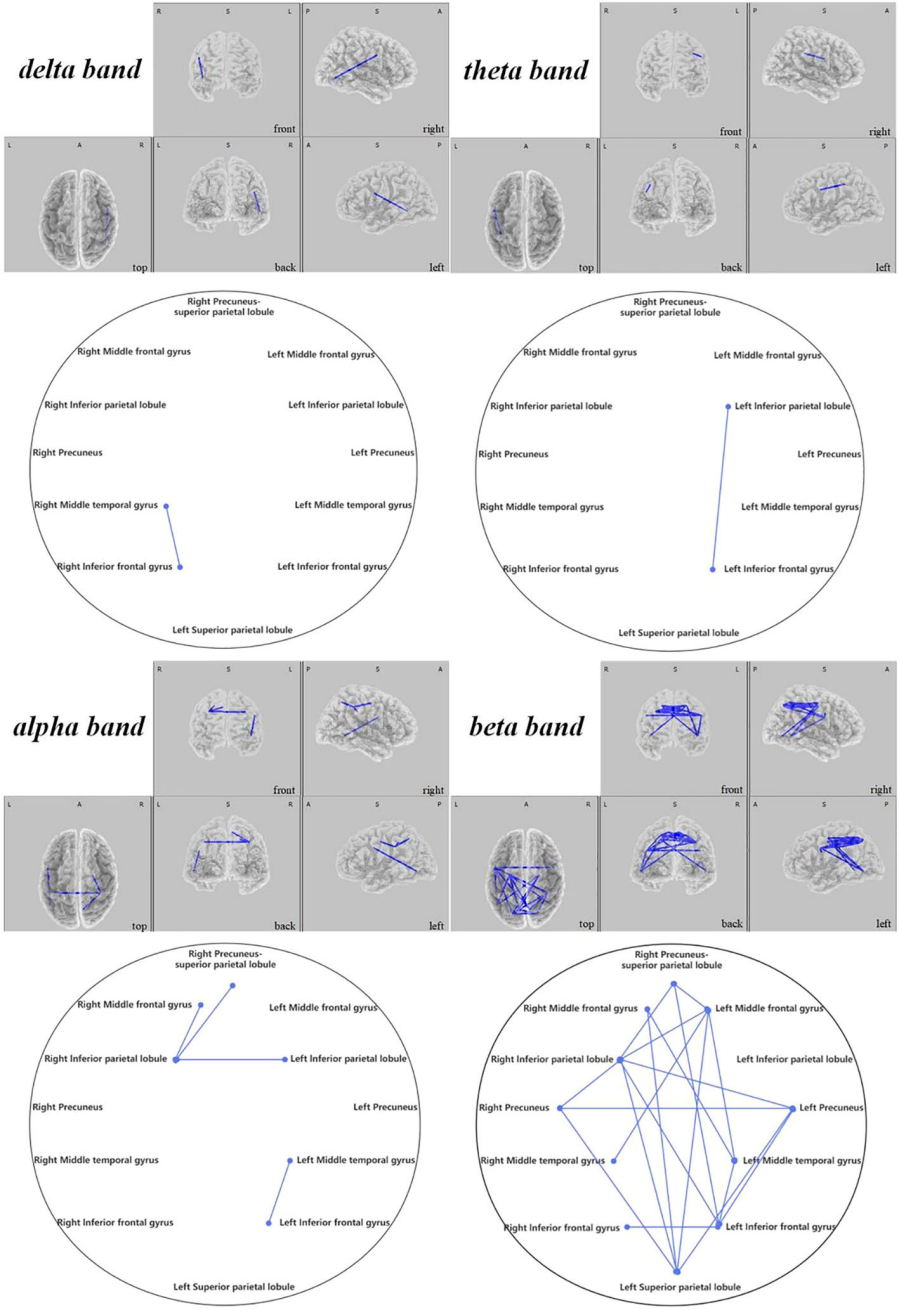
Connectivity variation diagram highlighting significant differences between pre-stimulation and post-stimulation groups. Blue lines represent a significant reduction in lagged phase synchronization within DAN for the pre-stimulation group as compared to the post-stimulation group. Orientational abbreviations indicate the following: L for left hemisphere, R for right hemisphere, A for anterior (frontal) regions, P for posterior (rear) regions, and S for superior (upper) regions of the brain.

In the active stimulation group, enhancements were observed in functional connectivity within brain networks post-intervention. The DMN displayed significant improvements particularly between the left superior temporal gyrus and medial frontal gyrus in the theta band ( t =-2.431, p< 0.048), and between the right superior temporal gyrus and medial frontal gyrus in the beta band ( t=−2.470 , p<0.036 ), as seen in **Figure 10**. Within the SMN, connectivity enhancements were primarily noted between the right precentral gyrus and right transverse temporal gyrus in the alpha band ( t =-2.704, p< 0.021), and numerous regions in the beta band as elaborated in **Figure 11**. The DAN showed marked improvements, especially between the right middle temporal gyrus and right inferior frontal gyrus in the delta band ( t =-2.626, p< 0.021), with additional connectivity augmentations displayed in various bands and areas as outlined in **Figure 12**. Contrarily, no statistically significant differences were reported in the sham stimulation cohort.

In examining the active stimulation group, our investigations illuminated significant findings within various neural networks of children with ASD, both preceding and following regulation. Our study’s salient results are elucidated as follows:

#### 3.2.1 Default mode network findings

In the DMN, we observed that ASD children exhibited significantly lower connectivity before regulation when compared to post-regulatory states. Notably, the connectivity rose notably between the left superior temporal gyrus and the medial frontal gyrus within the theta frequency band ( t =-2.431, p< 0.048). Furthermore, enhancements were apparent between the right superior temporal gyrus and the medial frontal gyrus in the beta frequency band ( t =-2.470, p< 0.036), as depicted in **Figure 10**.

#### 3.2.2 Sensorimotor network findings

Within the SMN, it was discerned that the brain connectivity in pre-regulation ASD children was significantly less than post-regulation. Such increments in connectivity manifested primarily between the right precentral gyrus and the right transverse temporal gyrus within the alpha frequency band ( t =-2.704, p< 0.021). Additionally, rising connectivity was noted in the beta band between the left transverse temporal gyrus and various other brain regions—namely the right precentral gyrus ( t =-3.657, p< 0.001), the left postcentral gyrus ( t =-3.353, p< 0.004), the medial frontal gyrus ( t =-3.858, p< 0.006), and the right transverse temporal gyrus ( t =-2.785, p< 0.021). Moreover, between the right precentral gyrus and the medial frontal gyrus, a significant increase in connectivity was observed ( t =-2.829, p< 0.014), presented in **Figure 11**.

#### 3.2.3 Dorsal attention network findings

Contrasts within the DAN revealed that ASD children’s pre-regulatory brain connectivity was substantially reduced compared to post-regulation. This was primarily seen between the right middle temporal gyrus and the right inferior frontal gyrus in the delta frequency band ( t =-2.626, p< 0.021) and between the left inferior parietal lobe and the left inferior frontal gyrus in the theta band ( t =-2.616, p< 0.024). In the alpha band, notable connectivity enhancements could be observed (1) between the right inferior parietal lobule and the right middle frontal gyrus ( t =-2.484, p< 0.04), the left inferior parietal lobule ( t =-3.645, p< 0.015), and the right precuneus-superior parietal lobule ( t =-3.143, p< 0.005), (2) as well as between the left middle temporal gyrus and the left inferior frontal gyrus ( t =-3.239, p< 0.001). Similarly, beta band findings revealed significant connections (1) between the right middle frontal gyrus and the left middle temporal gyrus (t=-2.960, p< 0.012), (2) bridging between the left and right precuneus ( t =-2.346, p< 0.048), (3) and from the left superior parietal lobule to the right middle frontal gyrus ( t =-2.555, p< 0.033), the left precuneus ( t =-2.683, p< 0.018), and the right precuneus ( t =-2.538, p< 0.039), (4) further linking the left middle frontal gyrus with the right inferior parietal lobule ( t =-3.171, p< 0.006).

## 4 Discussion

In this study, we evaluated the variations in current source density across the delta, theta, alpha, and beta bands within the brains of children diagnosed with ASD in comparison to typically developing children, as well as the changes in ASD children before and after undergoing tDCS stimulation. Utilizing lagged phase synchronization via sLORETA, we’ve examined functional connectivity alterations within three brain resting states before and after tDCS. Our findings indicated a notably lower current source density in the neural architecture of children in the ASD group when compared to the TD group. Post-tDCS regulation, the active stimulation group exhibited a substantial enhancement in brain current source density, which wasn’t observed in the sham stimulation group. Notably, ASD children’s brain activation improved significantly following tDCS intervention, with an increased number and distribution of voxels showing significant differences.

There is a well-documented array of evidence suggesting that individuals with ASD possess atypical brain structures and functioning (37–39), and non-invasive brain stimulation methods such as tDCS have demonstrated a considerable impact on modulating both brain activity and connective function (40, 41). Active tDCS stimulation, in contrast with sham, has been shown to reduce resting-state functional connections (42). Correspondingly, our study recorded a noteworthy rise in lagged phase synchronization within the DMN, SMN, and DAN in children with ASD post tDCS stimulation.

Existing research indicates that anomalous intrinsic functional connectivity of brain networks is a central aspect of social impairments in ASD (43). Moreover, the DMN—which plays a crucial role in processing self and other-related information and is implicated in recollecting episodic and autobiographical memories (44), as well as understanding others’ mental states (45)—has emerged as a pivotal system contributing to social deficits in ASD (46, 47). In line with this, our study discerned enhanced connectivity in the DMN between the medial frontal gyrus and bilateral superior temporal gyres in children with ASD post tDCS, which may significantly remodel internal information processing, potentially easing social impairments in ASD (48).

As a neural sensor, the SMN is primarily engaged in the handling and transmission of sensory and motor information. The interconnections between the different areas within this network ensure precise coordination of sensory and motor function. Diminished connectivity within the somatosensory and motor networks corroborates the atypical multisensorial and motor integration seen in ASD patients, likely contributing to core symptoms (49, 50). Our findings suggest that tDCS stimulation may elevate responsiveness to sensory input in individuals with ASD, thereby enhancing multisensory integration and consequently improving social capacities (51–53).

The DAN is recognized as a bilateral network that sustains attentional stability and contributes to the varying aspects of intelligence development (25, 54). Given the plethora of sensory signals the brain continually receives; DAN enables prioritization of attention to the foremost inputs at any given moment (55). Aberrations in DAN functional connectivity can manifest as impaired attention and hindered top-down processing in ASD. Following stimulation, increased connectivity within key DAN regions in our study might bolster attentional control and top-down goal-directed processing in individuals with ASD (25).

Thus, our research demonstrates that tDCS stimulation is safe and reliable, and may amplify brain activity and augment functional connectivity in individuals with ASD, potentially ameliorating core symptoms. And our study shows that tDCS can be applied to clinical treatment for a long time, and has far-reaching significance to improve the status quo of autistic children. Nevertheless, the employment of tDCS as a therapeutic approach for ASD remains in its nascent stages, with our study facing certain limitations. Firstly, our research depended on a relatively modest sample size with broader samples required in future inquiries. Additionally, evaluations were only conducted at two points—baseline and post-treatment. Subsequent research should incorporate multiple assessments, including follow-ups, to capture critical trajectories of tDCS’ effects. Thirdly, our study solely focused on the left DLPFC as a target area for tDCS, despite emerging studies that suggest various potential targets each potentially addressing specific ASD symptoms. Future investigations must explore these alternative sites. Lastly, while sLORETA serves as our means to measure brain activity and delineate regions, it is not without spatial limitations.

In conclusion, this study employed sLORETA to elucidate differences in brain activity between children with ASD and their TD counterparts, demonstrating that tDCS intervention in the DLPFC can enhance brain connectivity and evidencing the positive neuromodulatory effects of tDCS. Conferring the possibility that tDCS may emerge as a promising therapeutic avenue for children with ASD, its clinical application could become increasingly widespread, adding valuable practicality in clinical settings.

## Data availability statement

The raw data supporting the conclusions of this article will be made available by the corresponding author on reasonable request.

## Ethics statement

The studies involving humans were approved by the Ethics Committee of the Affiliated Hospital of Hebei University. The studies were conducted in accordance with the local legislation and institutional requirements. Written informed consent for participation in this study was provided by the participants’ legal guardians/next of kin.

## Author contributions

XL: Writing – review & editing, Funding acquisition, Conceptualization. JK: Writing – original draft, Supervision. YL: Writing – review & editing, Formal analysis, Data curation. SL: Writing – review & editing, Software, Investigation, Data curation. PH: Writing – review & editing, Supervision, Methodology.
